# Visible-wavelength two-photon excitation microscopy with multifocus scanning for volumetric live-cell imaging

**DOI:** 10.1117/1.JBO.25.1.014502

**Published:** 2019-11-05

**Authors:** Ryosuke Oketani, Haruka Suda, Kumiko Uegaki, Toshiki Kubo, Tomoki Matsuda, Masahito Yamanaka, Yoshiyuki Arai, Nicholas I. Smith, Takeharu Nagai, Katsumasa Fujita

**Affiliations:** a Osaka University, Department of Applied Physics, Suita, Osaka, Japan; b AIST-Osaka University, Advanced Photonics and Biosensing Open Innovation Laboratory, Suita, Osaka, Japan; c Osaka University, Institute of Scientific and Industrial Research, Ibaraki, Osaka, Japan; d Osaka University, Immunology Frontier Research Center, Suita, Osaka, Japan; e Osaka University, Institute for Open and Transdisciplinary Research Initiatives, Transdimensional Life Imaging Division, Suita, Osaka, Japan

**Keywords:** three-dimensional imaging, two-photon excitation, super-resolution microscopy, multifocus excitation, spinning disk confocal microscopy

## Abstract

Two-photon excitation microscopy is one of the key techniques used to observe three-dimensional (3-D) structures in biological samples. We utilized a visible-wavelength laser beam for two-photon excitation in a multifocus confocal scanning system to improve the spatial resolution and image contrast in 3-D live-cell imaging. Experimental and numerical analyses revealed that the axial resolution has improved for a wide range of pinhole sizes used for confocal detection. We observed the 3-D movements of the Golgi bodies in living HeLa cells with an imaging speed of 2 s per volume. We also confirmed that the time-lapse observation up to 8 min did not cause significant cell damage in two-photon excitation experiments using wavelengths in the visible light range. These results demonstrate that multifocus, two-photon excitation microscopy with the use of a visible wavelength can constitute a simple technique for 3-D visualization of living cells with high spatial resolution and image contrast.

Three-dimensional (3-D) observation of living biological samples has been recognized as an essential approach for the understanding of cellular functions because the 3-D distribution of intracellular molecules and their temporal dynamics are integral parts of biological activities.^1^ The morphological conditions in cell culture dishes are different from those of actual living organisms, which affects cell–cell communications, cell polarizations, and cellular morphology. Therefore, the sample preparation processes and the development of instrumentation have been studied in order to more accurately recreate and measure 3-D living samples.^2^^,^^3^

Thus far, several types of 3-D fluorescence imaging techniques have been developed for living cells and tissues. Light-sheet microscopy utilizes side illumination with a thin plane of light to illuminate only the focal plane of the objective lens for observation, thereby allowing 3-D imaging and significant reduction of photodamage and photobleaching in out-of-focus planes.^4^ The temporal resolution in light-sheet microscopy of over 1 billion voxels per min has also been demonstrated.^5^^,^^6^ In addition, the combined use of lattice light illumination has also been demonstrated in an effort to improve the spatial resolution of light-sheet microscopy to visualize the motion of finer intracellular structures.^7^ Other approaches used are based on laser scanning microscopy, such as confocal^8^ and two-photon excitation microscopy.^9^ With the use of a spinning disk for multifocus laser scanning, the temporal resolutions of confocal and two-photon excitation are respectively improved up to 1 ms per frame^10^ and 4.4 ms per frame,^11^ respectively. Combining spinning disk scanning and structured illumination can improve the efficiency of fluorescence detection,^12^ which is beneficial for reducing the photodamage in live-cell imaging. The spatial resolution improvement for multifocus microscopy is also demonstrated with the use of optical photon reassignment,^13^ image scanning microscopy,^14^^,^^15^ and instant structured illumination microscopy.^16^

In this study, we have demonstrated the use of visible wavelengths for two-photon excitation in multifocus scanning microscopy to improve the spatial resolution and image contrast in volumetric time-lapse imaging of living cells. Two-photon excitation with visible wavelength has been demonstrated by Drobizhev et al.^17^ and applied to super resolution microscopy by Yamanaka et al.,^18^ whereby the improvement of the spatial resolution and the image contrast were achieved by exploiting the nonlinear relation between excitation and emission. In the current research, we extended this concept with the implementation of a multifocus scanning system to apply v2PE microscopy to observe the 3-D dynamics of cellular components. We theoretically and experimentally evaluated the spatial resolution of the multifocus v2PE microscope. Time-lapse imaging of living HeLa cells in two-dimensional (2-D) and 3-D was performed with our technique with high temporal and spatial resolutions. We also evaluated photodamage during multifocus v2PE imaging to assess the prospects for long-time observation of living cells.

The experimental setup of multifocus v2PE microscopy is shown in Fig. 1. An infrared laser beam from a mode-locked Ti:sapphire laser (MaiTai HP, Spectra–Physics) was converted by an optical parametric oscillator (OPO, Inspire HF100, Spectra–Physics) to a visible-wavelength pulse beam with a pulse width of 200 fs, a repetition rate of 80 MHz, and the wavelength range of signal beam was tunable between 490 and 750 nm. The laser beam passed through a microlens array disk and a pinhole array disk equipped with a confocal scanning unit (CSU–X1–A–PTSP–2, Yokogawa) to form multifocus spots for fluorescence excitation. The multiple foci were imaged via a silicone–oil immersion objective to excite fluorescent probes in a sample that was placed on the stage of a commercial optical microscope (IX81, Olympus Corporation). Fluorescence from the sample was collected with the same objective and was delivered to the pinhole array disk that was responsible for the confocal effect. The fluorescence was separated from the excitation laser by a custom-made, long-pass dichroic mirror, with an edge wavelength of 506 nm, and was detected by an EM–CCD camera (ProEM:1024B–eXcelon–5, Princeton Instruments). By rotating the microlens array disk and the pinhole array disk at 4500 revolutions per minute (rpm), the fluorescence distribution of the sample was formed on the EM–CCD camera in 6.67 ms. A single-axis piezo stage (P–541.ZCL, PI) was used for axial scanning to obtain a 3-D distribution of fluorescence signals. We used two types of objective lenses: 100× (UPlanSApo 100×/1.35 Sil, Olympus Corporation) and 60× (UPlanSApo 60×/1.30 Sil, Olympus Corporation) by which the pinhole size equipped in the scanning system corresponded to 0.62 and 1.0 Airy units (AU), respectively, for the execution of the following experiments.

**Fig. 1:**
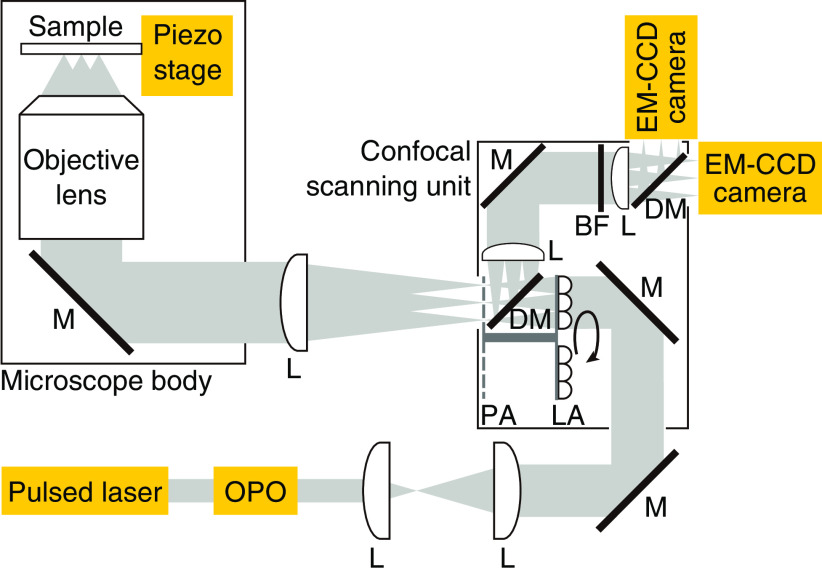
Optical setup of multifocus v2PE microscopy (OPO, optical parametric oscillator; L, lens; M, mirror; LA, lens array; DM, dichroic mirror; PA, pinhole array; BF, bandpass filter).

To confirm the spatial resolution of our setup experimentally, fluorescent beads with a diameter of 100 nm (FluoSpheres F8797, Thermo Fisher Scientific Inc.) were observed. The 100× objective lens with a numerical aperture (NA) of 1.35 was used for the observation. In our setup, the sizes of the pinholes were set to 0.62 AU because the use of smaller pinholes significantly decreases the fluorescence signal on the detector and makes it difficult to increase the temporal resolution. The wavelength and intensity for the excitation were 521 nm and $6.5\times 10^{4}\;W/{\mathrm{cm}}^{2}$, respectively. A total of 40 images were acquired at different sample depths for 3-D imaging with an exposure time of 5 s for each image. The pixel size for lateral images and the step size for axial scanning were set to 48 and 50 nm, respectively. Figure 2(a) shows the fluorescence image and its line profiles of a fluorescent bead. The full width at half maximum (FWHM) values of the image in the lateral and the axial directions are 177 and 297 nm, respectively. These values are close to the theoretical resolutions of the developed microscope as described in the following section.

**Fig. 2:**
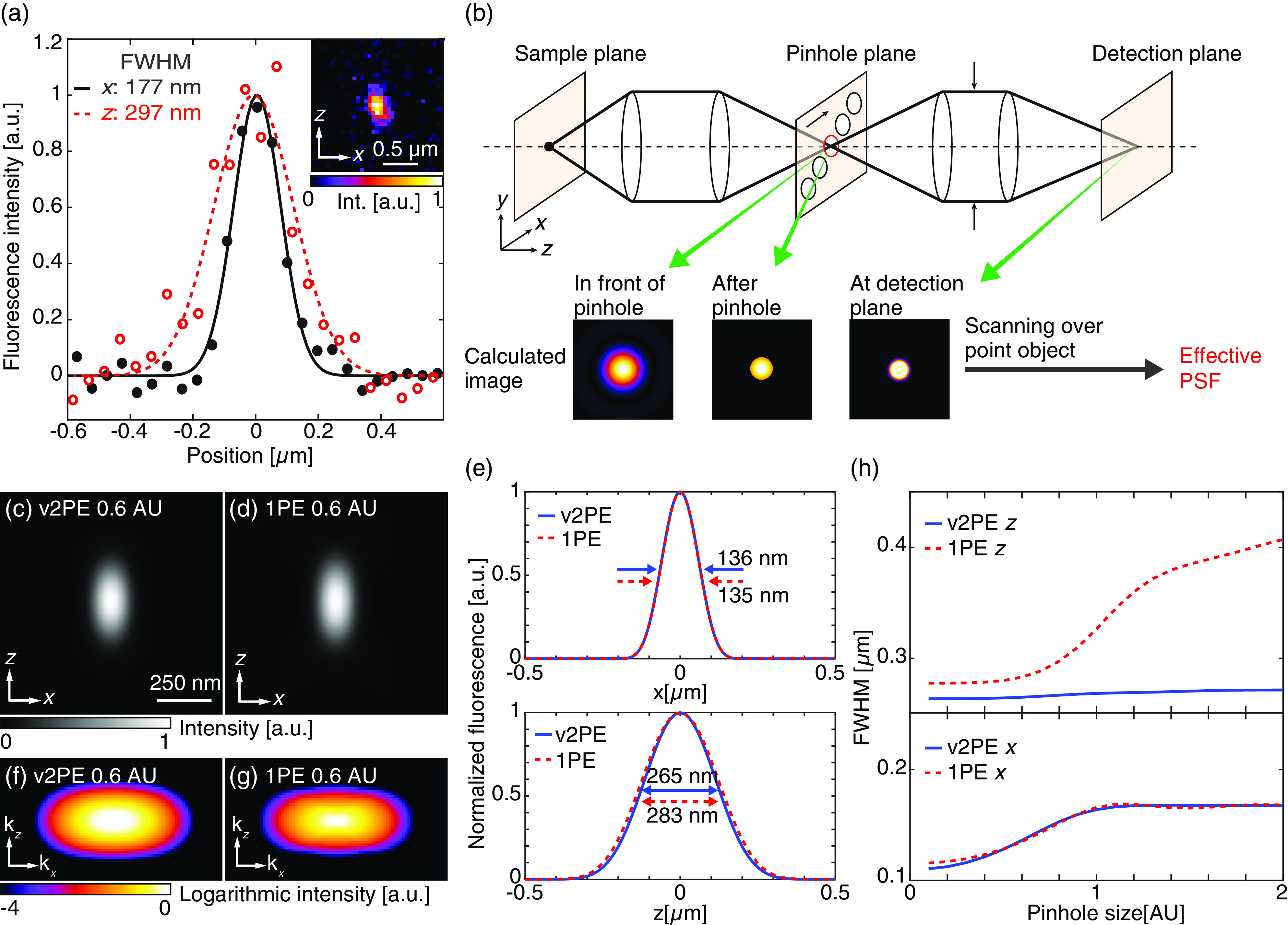
(a) Line profiles of the fluorescence image of a fluorescent bead (embedded). (b) Schematic explanation of the PSF and OTF calculation method in the detection path. (c), (d) Calculated PSFs of v2PE and 1PE multifocus microscopy. (e) Line profiles of (c) and (d) in x (upper) and z (lower) directions. (f), (g) Calculated OTF of v2PE and 1PE multifocus microscopy. (h) Relationship of pinhole size and FWHM of calculated PSFs in x (lower) and z (upper) directions.

We also investigated theoretically the spatial resolution of multifocus v2PE microscopy by simulating the image formation on an image sensor using two-photon excitation based on scanning foci. As shown in Fig. 2(b), unlike single-detector laser scanning microscopy, a fluorescence image was formed directly on the image sensor. Therefore, a fluorescence image was reconstructed as the summation of separate fluorescence images obtained from the scans of the different foci at different positions. Given that the cross talk between each focal spot was negligible in our setup, the intensity distribution formed by the focal spot of each beam on the image sensor was calculated individually. In this process, we also needed to estimate the diffraction of fluorescence light at the pinhole array, which affected the resultant spatial resolution of the microscope.

In the model, an infinitely small fluorescent object on the optical axis was illuminated at the focal spot. This provided different fluorescence intensities of the object, which depended on the position of the excitation focus. The fluorescence emission formed an optical image of the object in front of the pinhole that acted as a spatial filter and provided an input image to the imaging optics in the scanning unit. To simulate this, we applied a binary circular mask to the fluorescence image of the object and calculated the resultant image on the image sensor based on the theory of Fourier imaging, subject to the use of a spatial bandwidth, which was limited by the imaging optics. The point spread functions (PSFs) were calculated by summing the fluorescence images obtained by scanning the microlens and pinhole arrays at different excitation and corresponding pinhole positions.

Figures 2(c) and 2(d) show the calculated PSFs of a multifocus scanning system with v2PE at 521 nm and 1PE at 405 nm, respectively. The detected wavelength, NA of the objective, and the pinhole size for the calculation are set to 440 nm, 1.35, and 0.6 AU, respectively, which corresponds to our experimental condition. The line profiles of the calculated PSF are also shown in Fig. 2(e). By utilizing v2PE microscopy, the axial FWHM is decreased, thus indicating spatial resolution improvements even with the use of excitation wavelengths longer than 1PE. The lateral FWHM of v2PE is similar to that of 1PE and is caused by the accumulated detection in the multifocus system. A fluorescence distribution at the sample plane generated by the quadric response of 2PE is broadened at the detection plane by the linear imaging process of the detection path, thereby resulting in a similar spatial resolution to that of 1PE. Corresponding optical transfer functions (OTFs) are also shown in Figs. 2(f) and 2(g), thus indicating the improvement of axial resolution.

To understand the dependence of the FWHM of the PSF on the pinhole size, we also calculated the PSFs at different pinhole diameters in the range of 0.1 to 2.0 AU, as shown in Fig. 2(h). For the axial direction, the FWHM of v2PE is not affected by the pinhole diameter and is smaller than that of 1PE at any pinhole size as seen in the calculated OTF (see Fig. S1 in Supplementary Material). In the lateral direction, the FWHM of v2PE is similar to that of 1PE at pinhole sizes in the range of 0.4 to 2.0 AU. With a pinhole size smaller than 0.4 AU, the lateral spatial resolution of v2PE is higher than that of 1PE. Because the number of photons is significantly decreased for pinhole sizes smaller than 0.4 AU, it is not realistic to use such a small pinhole for live-cell imaging. Therefore, in practical condition, the lateral resolution of multifocus v2PE microscopy is essentially the same as that of multifocus microscopy with the 1PE.

To confirm the capability for time-resolved imaging of dynamic biological samples, we observed the Golgi bodies of living HeLa cells expressing a cyan fluorescent protein, mTFP1.^19^ The same objective lens was used for the measurements as that used for bead imaging (100×, NA1.35). The wavelength and intensity for the excitation were 530 nm and $1.86\times 10^{4}\;W/{\mathrm{cm}}^{2}$, respectively. 50 frames were measured at a frame rate of 1 frame per second (fps). For the wide-field imaging, the sample was illuminated by a mercury lamp with a wavelength range of 425 to 445 nm and an excitation intensity of $2.3\times 10^{-1}\;W/{\mathrm{cm}}^{2}$ for 1 s. The wavelength range of fluorescence detection was 460 to 510 nm. Figure 3(a) shows a respective frame from a fluorescence movie obtained with the use of our technique (see also Video 1) and Fig. 3(b) shows for comparison a fluorescence image acquired with a conventional wide-field microscope. The movements of individual Golgi bodies were resolved. Based on the comparison with the conventional wide-field image, the contrast of the image was improved because of the improvements of the spatial resolution and the depth discrimination capability. From the expanded frames shown in Figs. 3(c)–3(i), it was confirmed that several Golgi bodies appeared and disappeared during the observation period. This outcome is reasonable if we consider the movements of the Golgi bodies through the focal plane of the objective lens in the cytosol. The data further demonstrate the high-sectioning capability of v2PE microscopy. Based on prior reports that supported the fact that the use of v2PE can excite multiple fluorescent proteins with different emission wavelength simultaneously,^18^ our technique is applicable to the multicolor imaging of the 3-D dynamics of the intracellular molecules. We, therefore, also demonstrated multicolor imaging with an additional EM–CCD camera, a dichroic mirror (FF495–Di03 – 25×36), and an emission filter (FF02 – 460/80 – 25) on the detection path (Fig. 1). The Golgi bodies and fibrillarins in living HeLa cells were expressed with mTFP1 and EGFP, respectively. The detection ranges of each EM–CCD camera were 420 to 495 nm and 495 to 500 nm. The wavelength and intensity of the excitation were 525 nm and $7.5\times 10^{3}\;W/{\mathrm{cm}}^{2}$, respectively. A spectral unmixing method^20^ was executed to separate the fluorescence signals from each protein. As discussed in Ref. 18, the effect of autofluorescence is negligible in observation of living HeLa cells with the excitation wavelength used in this experiment. As shown in Figs. 3(j)–3(n), we also confirmed with multifocus scanning v2PE microscopy that the movements of the Golgi bodies were detected using multicolor imaging (Video 2).

**Fig. 3:**
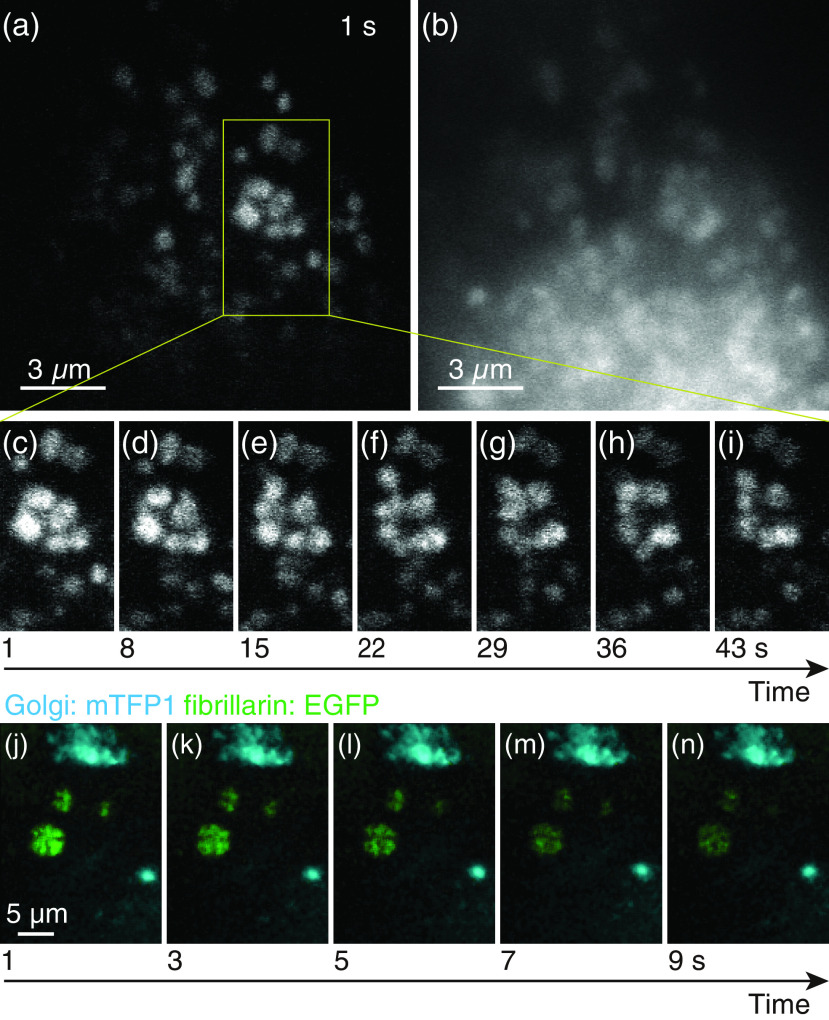
Fluorescence images of the Golgi bodies in a living HeLa cell. (a) Time-lapse v2PE microscopy movie (Video 1, QuickTime, 319 kB, https://doi.org/10.1117/1.JBO.25.1.014502.1). (b) Conventional wide-field microscopy. (c)–(i) A time sequence of the selected view in (a). No image filtering has been applied. (j)–(n) A time sequence of the time-lapse multicolor movie by v2PE microscopy (Video 2, QuickTime, 242 kB, https://doi.org/10.1117/1.JBO.25.1.014502.2). The mTFP1 (the Golgi body) and EGFP (fibrillarin) are colored as cyan and green, respectively. Spectral unmixing and median filter are applied to the raw images.

We also observed the 3-D structures and movements of the Golgi bodies in the living HeLa cells. For this observation, a silicone–oil immersion objective with a magnification of 60× was used. The frame rate of the 2-D image acquisition was set to 20 fps. Fluorescence images from 40 different layers in the sample were used for the formation of 3-D fluorescence volumes, with one volume taking 2 s to acquire, and then repeating to acquire 18 sequential volumes. The imaged volume size was 20×20×2  μm (xyz). The wavelength and intensity for excitation were 530 nm and $1.25\times 10^{5}\;W/{\mathrm{cm}}^{2}$, respectively. Figure 4 shows the result of the time-lapse 3-D observation of the Golgi bodies in a living HeLa cell (Video 3). The 3-D distribution of the Golgi bodies in the cell and their temporal dynamics are confirmed in Fig. 4.

**Fig. 4:**
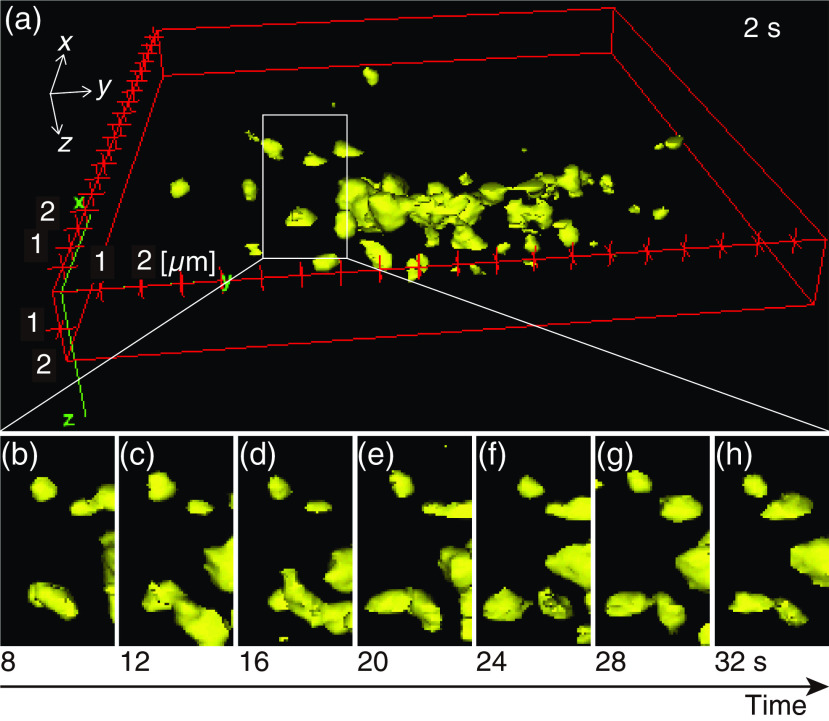
(a) The 3-D-rendered images of the Golgi bodies in a living HeLa cell obtained by time-lapse v2PE microscopy (Video 3, QuickTime, 15 MB, https://doi.org/10.1117/1.JBO.25.1.014502.3). The surface rendering has been produced by an ImageJ plugin, 3-D Viewer. (b)–(h) A time sequence of the clipped position in (a). A low-pass filter has been applied.

We also investigated the applicability of multifocus v2PE microscopy for the long-term observations of living cells. We used propidium iodide (PI) to indicate the cell damage after 7, 8, 9, and 10 min of time-lapse observation. For this evaluation, the sample was illuminated by 88 foci with an exposure time of 100 ms at 1-s intervals during the observations. The excitation intensity was $3.21\times 10^{4}\;W/{\mathrm{cm}}^{2}$, which was higher than the 2-D imaging in Fig. 3. The excitation wavelength was 525 nm, and a silicone–oil immersion objective with a magnification of 60× was used. As shown in Figs. 5(a)–5(d), we have confirmed the introduction of PI into the cells with an observation period longer than 9 min. The phase contrast images shown in Figs. 5(e)–5(h) also confirmed the bubbling effect in the sample. With the excitation conditions that used an exposure time of 500 ms at 5-s intervals, we also confirmed the PI introduction with an observation period longer than 9 min. From these experiments, the time-lapse observations can be conducted without inflicting visible cell damage during periods shorter than 8 min. Note that for 3-D time-lapse imaging, we can expect prolonged cell viability because the focal plane for excitation changes during the scanning of the sample along the axial direction.

**Fig. 5:**
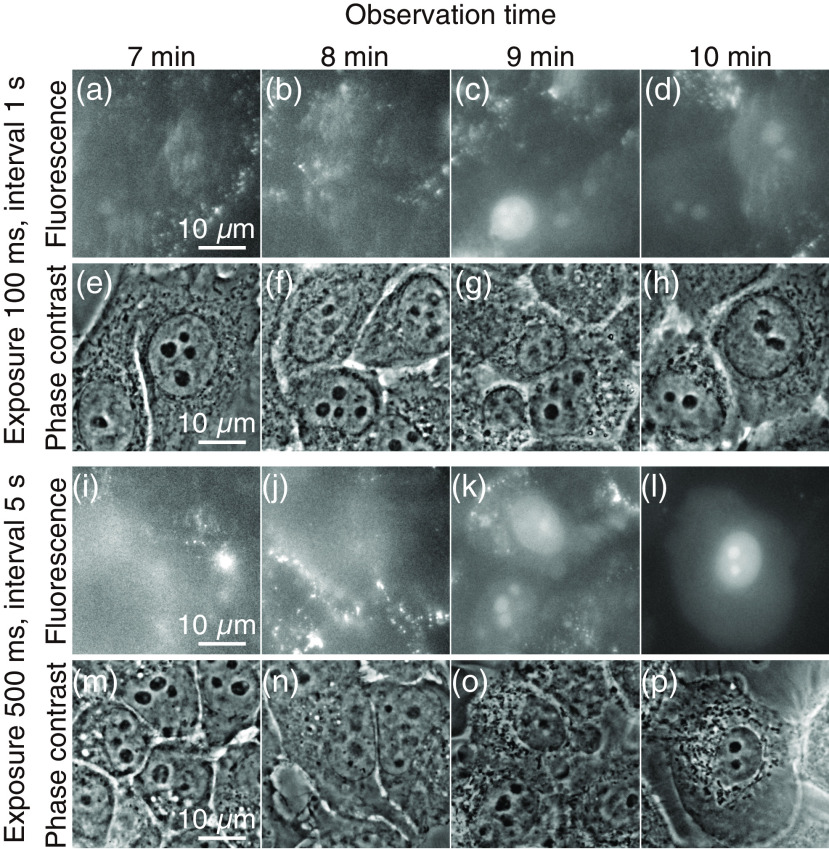
(a)–(d), (i)–(l) Fluorescence images of HeLa cells treated with PI. (e)–(h), (m)–(p) Phase contrast images corresponding to (a)–(d) and (i)–(l), respectively. The total observation time is 7 min for (a), (e), (i), and (m); 8 min for (b), (f), (j), and (n); 9 min for (c), (g), (k), and (o); and 10 min for (d), (h), (l), and (p). The exposure time and the interval of the exposure are 100 ms and 1 s for (a)–(h) and 500 ms and 5 s for (i)–(p), respectively.

In this research, we developed a multifocus microscope using visible-wavelength, two-photon excitation to improve the spatial resolution in volumetric live-cell imaging. Especially for 3-D biological imaging, the axial resolution is important, and from our observations of a fluorescent bead, FWHM values of 177 and 297 nm were achieved in the lateral and axial directions, respectively, which were far better than the axial resolution of multifocus 1PE microscopy. This spatial resolution is also comparable with that of 3-D lattice light-sheet microscopy.^7^ Although the field of view of our technique was relatively smaller than the lattice light-sheet technique, our technique can achieve a similar resolution with a simple instrumentation setup with a single objective lens for both illumination and detection.

In our experiments, the temporal resolution has been mainly determined by the limitation of the laser power that was available from the OPO. A light source with a higher output power can improve the imaging speed. Although the v2PE excitation leads to spatial resolution improvements in a single-point scanning system, multifocus v2PE microscopy has a lateral resolution identical to that of 1PE multifocus microscopy. This is due to the difference in the imaging formation between the multifocus and the single-point scanning techniques. Because the excitation volume of v2PE is smaller than that of 1PE, it is possible to improve the spatial resolution further by introducing the image scanning technique.^16^^,^^21^ This technique can be realized by introducing an additional microlens array in front of the pinhole array.^13^

We have confirmed sample damage when a single plane is illuminated repeatedly over 9 min, which can be an issue for long-term imaging. For 3-D imaging, however, axial scanning of samples can avoid continuous excitation of an observing layer, thus resulting in the longer observation time. Therefore, our technique can be applied to time-lapse 3-D imaging for more than 9 min by adjusting the illumination conditions. Although the sample damage associated with the use of other techniques, such as light-sheet microscopy, can be less extensive than our technique, our technique can visualize cellular functions in a cell, such as the release of calcium ions and vesicular transport with high-image contrast by combining visible-wavelength, two-photon excitation, simultaneous excitation of multiple fluorophores,^18^ and the use of functional fluorescent probes.^22^ Photobleaching can also be the issue because of the resonance effect at the visible region. It is important to optimize the excitation intensity and exposure time for longer observation.^18^
