# Supplementary material for: Visible-wavelength two-photon excitation microscopy with multifocus scanning for volumetric live-cell imaging
Source: J Biomed Opt. 2019 Nov 5;25(1):014502. doi: 10.1117/1.JBO.25.1.014502 (PMC7008499; doi:10.1117/1.JBO.25.1.014502)
Supplement: Supplementary file 1 [file JBO_025_014502_SD001.pdf]

Visible-wavelength two-photon excitation microscopy with multifocus scanning for volumetric live-cell imaging

Ryosuke Oketani, Haruka Suda, Kumiko Uegaki, Toshiki Kubo, Tomoki Matsuda, Masahito Yamanaka, Yoshiyuki Arai, Nicholas I. Smith, Takeharu Nagai, Katsumasa Fujita

Supplemental Figure (Fig. S1)

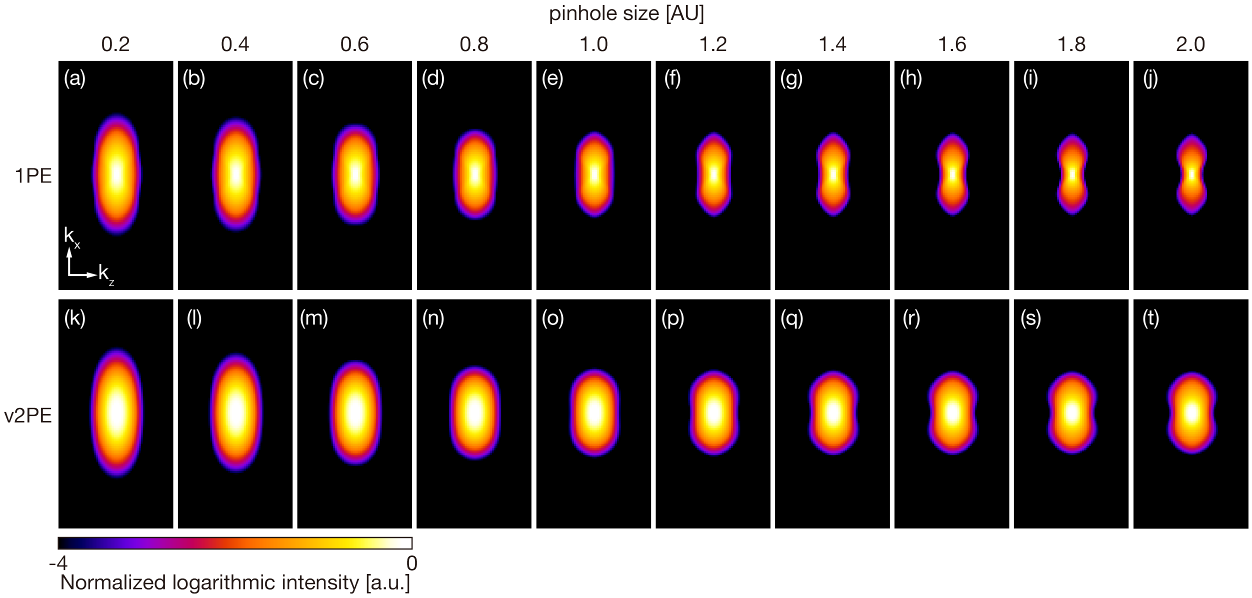

Fig. S1 Calculated results of normalized OTF for multifocus confocal microscopy with pinhole sizes in the range of 0.2 to 2.0 AU and a pitch of 0.2 AU. (a–j) 1PE and (k–t) v2PE. The color bar shows a normalized logarithmic intensity scale. The other parameters used for calculations are the same as those used in the main text.
